# Simulated directed-learning in life-education intervention on the meaning of life, positive beliefs, and well-being among nursing students: A Quasi-experimental study

**DOI:** 10.1097/MD.0000000000016330

**Published:** 2019-07-05

**Authors:** Fu-Ju Tsai, Yih-Jin Hu, Cheng-Yu Chen, Gwo-Liang Yeh, Chie-Chien Tseng, Si-Chi Chen

**Affiliations:** a Department of Health Promotion and Health Education, National Taiwan Normal University, Taiwan R.O.C.; b MSN, Department of Nursing, Emory University, GA; c Department of Nursing, Fooyin University, Taiwan R.O.C.; d University of Florida, FL; e Department of Education, National Taipei University of Education, Taiwan R.O.C.

**Keywords:** health education, health promotion, meaning of life, nursing students, positive beliefs, well-being

## Abstract

Nursing educators have the responsibility to equip nursing students with knowledge about the meaning of life, positive beliefs, and well-being in order to enhance their physical, psychological, spiritual, and social health education and promotion. The purpose of this study was to explore nursing students’ simulated directed-learning in a life-education intervention on the meaning of life, positive beliefs, and well-being in regard to immediate and delayed effects in improving physical, psychological, spiritual, and social health education and promotion.

The method of this study was constituted a quasi-experimental design with experimental and control groups for pre-test, post-test, and post-post-test. Purposive sampling and non-random distribution were used in the study. Assigned to the experimental group, 54 participants were third-year nursing students enrolled in a health education course with simulated directed-learning in a life-education intervention. Assigned to the control group, 56 participants were third-year nursing students enrolled in a caring care course without simulated directed-learning in a life-education intervention. A 56-item questionnaire was utilized, and the content validity index (CVI) was 0.95, as determined by seven expert scholars. The reliability of the questionnaire (n = 45) on Cronbach's α were: meaning of life 0.96, positive beliefs 0.95, and well-being 0.96. The statistical package SPSS 23.0 was used to analyze all of the data in the study. Frequencies, percentages, pre-test mean and SD, post-test mean and SD, post-post-test mean and SD, chi-squared test, *t* test, and generalized estimating equation (GEE) were employed for data analysis.

Nursing students in the experimental group compared with the control group exhibited significant differences in meaning of life on the pre-post-test (β = 16.40, *P* < .001) and pre-post post-test (β = 25.94, *P* < .001), positive beliefs on the pre-post-test (β = 5.64, *P* < .01) and pre-post post-test (β = 9.21, *P* < .001), and well-being on the pre-post-test (β = 14.33, *P* < .001) and pre-post post-test (β = 23.68, *P* < .001).

Nursing students in the experimental group showed a significant improvement in the simulated directed-learning with a life-education intervention on meaning of life, positive beliefs, and well-being in the immediate and delayed effects that enhanced their physical, psychological, spiritual, and social health education and promotion.

## 1 Introduction

In 1998, according to the World Health Organization (WHO), health education and promotion comprise the following four aspects: physical, psychological, spiritual, and social health.^[1]^ Since health education and promotion are the major health concepts for nursing students in a health education course, nursing educators need to develop nursing students’ health literacy in health education and promotion.^[2]^ Regarding positive and negative health concepts, nursing educators design health education and promotion to optimally develop nursing students’ health skills in nursing education.^[3,4]^ Currently, the Internet, websites, e-books, YouTube videos, and e-movie-based healthcare information are positively correlated with e-learning to develop nursing students’ health skills and to promote a safe-learning environment.^[5]^ Therefore, nursing educators may improve nursing students in physical, psychological, spiritual, and social health education, including meaning of life, positive beliefs, and well-being with e-learning health information in the teaching and learning process.^[6]^

Meaning of life is related to love, hope, honesty, kindness, gratitude and social intelligence, ^[7]^ and constitutes a protective factor against depression, hopelessness, and suicidal ideation^[8]^ in terms of mental health.^[9]^ Spiritual health is positively associated with life orientation and psychological properties,^[10]^ while psychosocial health is positively correlated with well-being related to life meaning, life satisfaction, religious meaning, spirituality, social support, and quality of life.^[11]^ Meaning of life also increases the sense of life fulfillment for dying people to achieve life satisfaction, social support, and quality of life.^[12]^ Therefore, meaning of life is linked with psychological well-being and mental health education and promotion^[13]^ to establish many goals of lifestyle change for improved quality of life.^[14]^ Moreover, positive beliefs are essential for people to manage and adapt to errors in the learning process for improving their quality of life.^[15]^ People who hold positive beliefs that are associated with disease-treatment outcomes are more likely to achieve a higher quality of life.^[16]^ Positive beliefs can also increase a person's values and mental health to cope with negative emotional changes^[17]^ and assist in life satisfaction among students for a better quality of life.^[18]^ Therefore, positive beliefs include optimism and life satisfaction in pursuing successful health aging for future quality of life.^[19]^ Furthermore, well-being is positively correlated with positivity, humor, life satisfaction, empowerment, social connections, and emotional self-efficacy to obtain a high quality of life.^[20]^ Psychological and physical health are also positively correlated with managing depression and obtaining psychological well-being.^[21]^ Well-being is an important element for nursing students’ quality of life. Promoting nursing students’ emotional well-being is positively associated with psychological well-being, social health education, quality of life, and enjoyable learning in the classroom.^[22]^

Health education and promotion interventions are associated with life situations to improve quality of life and attain productive learning in the classroom, and thus nursing educators consider many teaching methods that can enhance nursing students’ physical, psychological, spiritual, and social health education and promotion of the meaning of life^[23]^, positive beliefs, and well-being.^[24]^ For example, simulated directed-learning is an effective teaching method to equip nursing students’ learning with health knowledge, attitudes, and behaviors for a better quality of life.^[25]^ In Taiwan, nursing students tend to have improper diets, lack exercise, smoke tobacco, drink alcohol, and exhibit poor moods, negative thinking, stress, and depression.^[26]^ Overwork is also a common condition among nurses in Taiwan, which leads to major health problems, such as physical illnesses, emotional control difficulties, cancer, sudden death, psychological depression, and suicide. Many nursing students have depression associated with personal, family, school, and other factors.^[26]^ Nursing students could possibly have health problems resulting from a lack of the meaning of life, positive beliefs, and well-being to negatively impact physical, psychological, spiritual, and social health education and promotion.

The motivations of this study were to integrate simulated directed-learning in a life-education intervention in a health education course and improve nursing students concerning the meaning of life,^[27,28]^ positive beliefs,^[29,30]^ and well-being^[31,32,33]^ with physical, psychological, spiritual, and social health education and promotion.

## 2 Purpose

The purpose of study was to explore nursing students’ simulated directed-learning in a life-education intervention on the meaning of life, positive beliefs, and well-being as assessed by corresponding immediate and delayed effects.

## 3 Methods

### 3.1 Design

This study adopted a quasi-experimental design with an experimental group and a control group for pre-test, post-test, and post-post-test.

### 3.2 Framework

**Figure 1:**
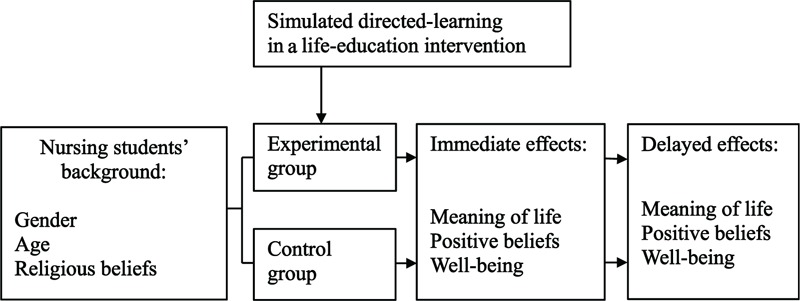
The framework of this study.

The framework of this study was as follows (Fig. 1): nursing students’ background included gender, age, and religious beliefs. The participants in the study were nursing students assigned to either an experimental group or a control group. Nursing students in the experimental group received simulated directed-learning in a life-education intervention to promote meaning of life, positive beliefs, and well-being in immediate (after intervention) and delayed effects (4 weeks after the intervention) (Fig. 1). Nursing students in the control group received no simulated directed-learning in a life-education intervention to improve meaning of life, positive beliefs, and well-being in the immediate (after non-intervention) and delayed effects (4 weeks after the non-intervention) (Fig. 1).

### 3.3 Participants

The G-Power estimated value was utilized to determine how many participants were needed for this study, set up *t* tests, and select means: difference between independent means (2 groups), using 2 tails, effect size = 0.8, α = 0.05, and power = 0.8. The G-Power system indicated the need for 52 participants: 26 participants in the experimental group and 26 in the control group. In order to avoid missing participants, the researchers recruited twice the number of required participants in the study. A purposive sample, including 114 participants was recruited in this study. There was a non-random distribution for the experimental and control groups. Fifty-five third-year nursing students enrolled in a health education course were assigned to the experimental group, and 59 third-year nursing students enrolled in a caring care course were assigned to the control group. One-hundred-fourteen participants voluntarily completed questionnaires three-times in the pre-test, post-test, and post-post-test, respectively. Ultimately, 110 (96.49%) participants were included 54 (98.18%) in the experimental group and 56 (94.92%) in the control group. All participants completed the 3 questionnaires on the pre-test, post-test, and post-post-test.

### 3.4 Ethical considerations

This study was approved by the Institutional Review Board of Yuan's General Hospital (IRB No. 20171130B) in Taiwan, R.O.C. After completion of the study, the researcher provided a three-part Power Point presentation on the meaning of life, positive beliefs, and well-being for nursing students in the control group, and also included the presentation on an e-learning platform of a University.

### 3.5 Simulated directed-learning in a life-education intervention

A nursing educator designed three lectures (each week/each time lasting 100 min for three continuous weeks, totaling 300 min) on simulated directed-learning with a life-education intervention on three topics: the meaning of life, positive beliefs, and well-being in a health education course in which teaching and learning contents were linked to YouTube videos, e-books, and Internet movies materials (Table 1). Nursing students were able to learn the three topic contents in the classroom and download them from an e-learning platform at a future time.

**Table 1:**
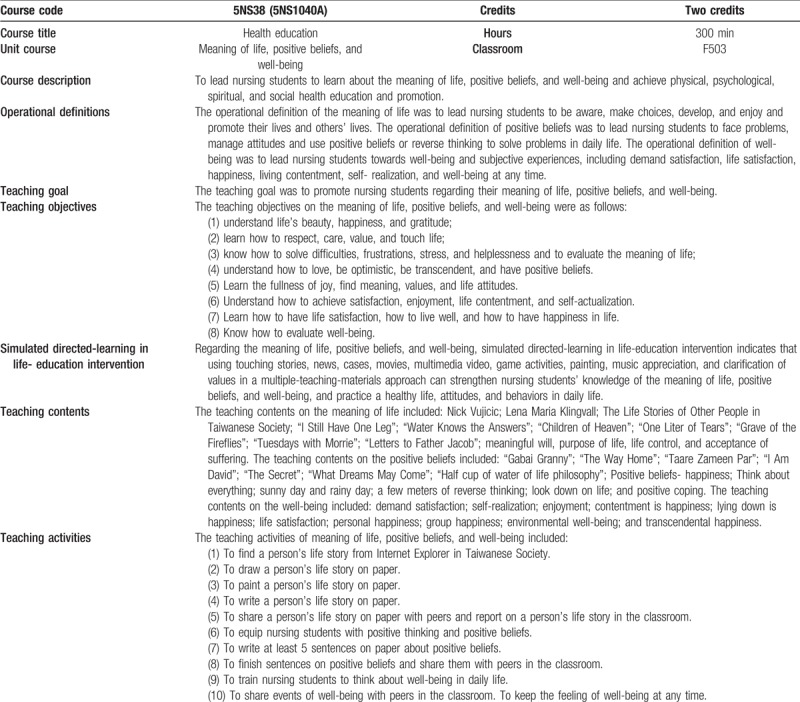
Teaching plan on the meaning of life, positive beliefs, and well-being.

A life-education intervention on the meaning of life, positive beliefs, and well-being can equip nursing students with competencies to take care of patients in the clinical workplace. Many evidence-based researches support the effectiveness of life-education interventions. Meaning of life is effective for the blind or visually impaired to accept their physical and psychological condition.^[34]^ The core values of life are related to self-confidence, resolution, mental activities, reflecting on life, expanding life, solitary spirit, self-awareness, life experience, and meaning of life.^[35]^ Life education may train students in problem solving, reaching life goals, and establishing moral values.^[36]^ Many students who take a life education course then go on to have a joyful life, a strong meaning of life, and establish knowledge and social spirit.^[37]^ Integrating life education into picture books for language teaching has been shown to positively affect the empathy, self-esteem, and emotional management of many students.^[38]^

In addition, simulated directed-learning achieves better learning effects than facilitator training. Indeed, many students obtain major positive learning effects from simulated directed-learning, including learning knowledge, motivation, operational skills, and competencies.^[39]^ After learning basic life skills over three months, nursing students increased learning of their life support competencies.^[39]^ Simulated-based education with combined team-based learning constitutes an effective teaching method for nursing students.^[40]^ One research explored nursing students’ physical assessment in self-directed learning in order to increase nursing competences, learning satisfaction, and self-confidence.^[41]^ Self-directed learning with an ultrasound simulator guide has been demonstrated to be a superior teaching model compared to the traditional course model.^[42]^ Simulated teaching may also augment healthcare providers’ nursing competencies and self-confidence, while decreasing stress in the clinical workplace.^[43]^

### 3.6 Instruments

The instruments of this study were taken from the Life Attitude Profile by Ho^[44]^ and the Positive Coping, Spirituality and Well-Being Scale by Lin and Yu.^[45]^ A 56-item questionnaire was used to explore nursing students views on meaning of life (1–25 items), positive beliefs (1–11 items), and well-being (1–20 items) in terms of the immediate and delayed effects for improving physical, psychological, spiritual, and social health education and promotion. The questionnaire included nursing students’ academy, department, subject, gender, age, religious beliefs, meaning of life, positive beliefs, and well-being. A 5-point Likert, scale ranging from “completely disagree” 1 to “completely agree” 5 was used for this study. The content validity index (CVI) of the questionnaire was established at 0.95 by 7 expert scholars. The reliability of the questionnaire (n = 45) on Cronbach's α was meaning of life 0.96, positive beliefs 0.95, and well-being 0.96.

### 3.7 Data collection

The researcher administered questionnaires three times, that is, once each on the pre-test, post-test, and post-post-test to all nursing students. All nursing students were then informed that these questionnaires aimed to investigate nursing students’ views of the meaning of life, positive beliefs, and well-being to augment physical, psychological, spiritual, and social health education and promotion. All of the nursing students in the experimental and control groups could decide to completely or incompletely fill out questionnaires on the pre-test, post-test, and post-post-test. All 110 questionnaires of the experimental and control groups were completely finished (96.49%) with four outflowed (3.51%). The researcher collected all completed 54 (98.18%) questionnaires by the experimental group and 56 (94.92%) questionnaires by the control group for the pre-test, post-test, and post-post-test from May 1 to June 21, 2018.

### 3.8 Data analysis

The statistical package SPSS 23.0 was used to analyze all of the data in the study. Frequencies, percentages, pre-test mean and SD, post-test mean and SD, post-post-test mean and SD, chi-squared test, *t* test, and generalized estimating equation (GEE) were utilized for data analysis in the study.

## 4 Results

### 4.1 Nursing students’ distribution in the experimental and control groups

Nursing students’ distribution, including 54 in the experimental group and 56 in the control group were not significantly different regarding gender, age, and religious beliefs (Table 2). In terms of gender distribution, nursing students comprised 6 (11.10%) males and 48 (88.90%) females in the experimental group and comprised 4 (7.10%) males and 52 (92.90%) females in the control group (Table 2). Concerning age distribution, nursing students included 15 (27.78%) 17-year-olds, 32 (59.26%) 18-year-olds, 3 (5.56%) 19-year-olds, and 4 (7.40%) 20-year-olds in the experimental group, and included 15 (26.80%) 17-year-olds, 39 (69.60%) 18-year-olds, and 2 (3.60%) 19-year-olds in the control group (Table 2). Regarding the distribution of religious beliefs, nursing students included 21 (38.90%) with no religious beliefs, 6 (11.10%) Christians, 9 (16.70%) Buddhists, and 18 (33.30%) Taoists in the experimental group and included 29 (51.80%) with no religious beliefs, 2 (3.60%) Christians, 1 (1.80%) Catholic, 6 (10.70%) Buddhists, and 18 (32.10%) Taoists in the control group (Table 2).

**Table 2:**
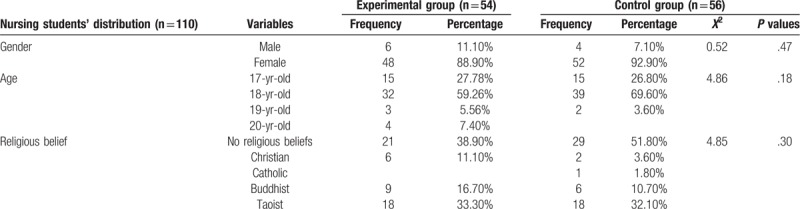
Nursing students’ distribution in the experimental and control groups.

### 4.2 *T* test (pre-test) on the meaning of life, positive beliefs, and well-being

In the *t* test analysis on the pre-test, all nursing students in the experimental and control groups showed significant differences on meaning of life (*P* < .01), no significant differences on positive beliefs, and significant differences on well-being (*P* < .001) (Table 3).

**Table 3:**
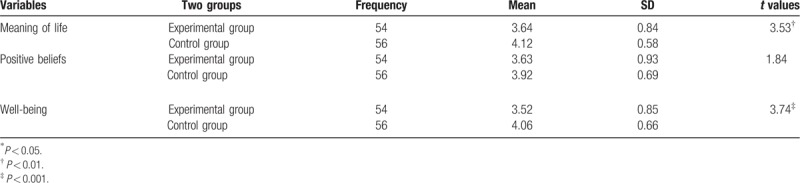
*T* test (pre-test) on the meaning of life, positive beliefs, and well-being.

### 4.3 Comparison of the experimental and control groups on the mean scores of meaning of life, positive beliefs, and well-being

The experimental group and control group exhibited significant differences on the mean scores of meaning of life, positive beliefs, and well-being. In terms of meaning of life, the experimental group showed a comparative mean score on the pre-test of −11.84 (SD 3.20), post-test of 4.56 (SD 3.08), and post-post-test of 14.09 (SD 3.16) (Table 4). Regarding positive beliefs, the experimental group indicated a comparative mean score on the pre-test of −3.11 (SD 1.60), post-test of 2.53 (SD 1.49), and post-post-test of 6.10 (SD 1.45) (Table 4). Concerning well-being, the experimental group showed a comparative mean score on the pre-test of −10.73 (SD 2.71), post-test of 3.60 (SD 2.66), and post-post-test of 12.94 (SD 2.52) (Table 4).

**Table 4:**
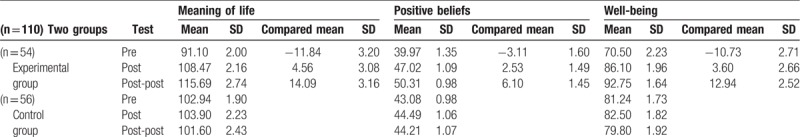
Comparison of the experimental and control groups on the mean scores of meaning of life, positive beliefs, and well-being.

### 4.4 GEE analysis of the experimental and control groups on the meaning of life, positive beliefs, and well-being

The GEE analysis revealed that all of the nursing students in both groups had significant differences on meaning of life on the pre-post-test (β = 16.40, *P* < .001) and pre-post post-test (β = 25.94, *P* < .001), positive beliefs on the pre-post-test (β = 5.64, *P* < .01) and pre-post post-test (β = 9.21, *P* < .001), and well-being on the pre-post-test (β = 14.33, *P* < .001) and pre-post post-test (β = 23.68, *P* < .001) (Table 5). The experimental group exhibited significant improvement on meaning of life, positive beliefs, and well-being compared to the control group.

**Table 5:**
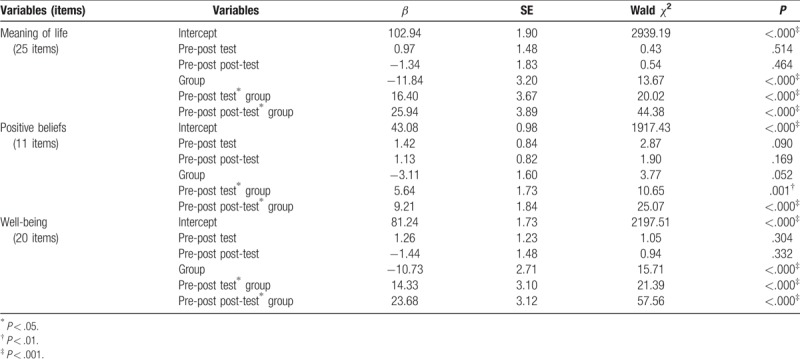
GEE analysis of experimental and control groups on the meaning of life, positive beliefs, and well-being.

## 5 Discussion

The results of the present study demonstrate that simulated directed-learning with a life education intervention develops meaning of life,^[47]^ positive beliefs,^[48]^ and well-being,^[46,49,50]^ and augments physical, psychological, spiritual, and social health education and promotion among nursing students for an improved quality of life. The quality of simulation activities in nursing education augments nursing students’ clinical skills,^[51,52]^ self-efficacy and critical thinking skills,^[53]^ and provides an improved quality of life to many patients in clinical settings. Furthermore, a situation simulation is used for problem-solving in daily life^[54]^ that is related to teaching and learning processes for nursing students to learn health education and promotion in a nursing education course. The strategy of situated simulation aims to improve nursing students’ handling of stress and fear in clinical practice and to increase professional skills.^[55]^ The special simulation is to provide the same quality end-of-life education and life experiences for nursing students in health education and promotion.^[56]^ Therefore, simulated directed-learning materials with many situations for nursing students in a health education course aimed to continue to train nursing students on an e-learning platform with a life-education intervention on the meaning of life, positive beliefs, and well-being for a better quality of life.

In this study, simulated directed-learning constituted an effective method to raise nursing students’ meaning of life, positive beliefs, and well-being in a health education course. These results were in accordance with those in the extant literature on medical education, nursing care, health promotion, interpersonal interaction, and other educational fields. Indeed, many articles document to show on the teaching and learning effects in relation to evidence-based practice with nursing students’ simulated directed-learning on health knowledge, attitudes, behaviors, nursing skills, critical thinking, problem-solving, self-efficacy, many competencies,^[51,53]^ meaning of life, positive beliefs, and well-being. Nursing Students’ meaning of life and well-being can predict 79% of their positive beliefs.^[57]^ Therefore, this study agrees with numerous other articles in using simulated-directed learning with a life education intervention to improve nursing students’ meaning of life, positive beliefs, and well-being in relation to physical, psychological, spiritual, and social health education and promotion.

A unique finding of the present study was that all nursing students in the experimental and control groups on the pre-test analysis showed significant differences on meaning of life, no significant differences on positive beliefs, and significant differences on well-being. Because the experimental and control groups were purposive samples with a non-random distribution in the study, 2 groups indicated significant differences on meaning of life and well-being on the pre-test analysis. Therefore, the data indicated significant differences in both groups on meaning of life and well-being with pre-test on the *t* test analysis, but it did not impact the results of this study on meaning of life, positive beliefs, and well-being on the post-test and post-post-test.

Most studies in the general areas of health education and promotion have not identified significant differences in *t* test analysis in a pre-test in experimental and control groups. One study, however, offered a quasi-experimental design that was related to peer learning for the practice of nursing skills in communication, cooperation, reflection and independence, and to improve nursing students’ self-efficacy.^[58]^ A quasi-experimental study explored a training design, in which positive and negative effects were related to the development of managerial sensing.^[59]^ Positive behavior interventions with a quasi-experimental design have also been demonstrated to increase students’ meaningful improvement.^[60]^ Another study employed a pre-post quasi-experimental design with nursing students who received writing training to increase their competencies in efficacy and effectiveness within the nursing classroom.^[61]^ The above research showed that experimental and control groups exhibited no significant differences on *t* test (pre-test) analysis. Therefore, this research differs from other research in pre-test and *t* test analysis for experimental and control groups.

Moreover, nursing students in the experimental group concerning meaning of life indicated a mean score on the pre-test of −11.84; positive beliefs indicated a mean score on the pre-test of −3.11; and well-being indicated a mean score on the pre-test of −10.73 compared to the control group. The experimental group's pre-test mean score on meaning of life, positive beliefs, and well-being were lower than the mean scores of the control group. However, the experimental group, which received simulated directed-learning in a life-education intervention on the meaning of life, positive beliefs and well-being, had higher mean scores on the post-test and post-post-test than did the control group. Moreover, nursing students in the experimental group on meaning of life indicated a mean score on the post-test of 4.56 and post-post-test of 14.09; positive beliefs indicated a mean score on the post-test of 2.53 and post-post-test of 6.10; and well-being indicated a mean score on the post-test of 3.60 and post-post-test of 12.94 compared to the control group. Therefore, the results of the present study were in accordance with other evidence-based research. Simulated directed-learning on the meaning of life, positive beliefs, and well-being constituted an effective teaching and learning process to change lifestyles in terms of nursing students’ physical, psychological, spiritual, social health education and promotion for an improved quality of life.^[62,63]^

In addition, the experimental and control groups exhibited significant differences on the pre-test analysis on meaning of life and well-being, but not on positive beliefs. Consequently, the researcher used GEE analysis to explore both groups, and found significant differences on meaning of life, positive beliefs, and well-being on the post-test and post-post-test. A previous study utilized GEE analysis to evaluate teaching and learning effects on medical education that was related to positive impact on students’ medication knowledge, efficacy, and behavior .^[64]^ The study indicated that the experimental group had significant differences on meaning of life, positive beliefs, and well-being on the pre-post-test and pre-post post- test in comparison with the control group. Therefore, nursing students in the experimental group with simulated directed-learning in a life education intervention showed significant improvement in meaning of life, positive beliefs, and well-being with immediate and delayed effects compared to the control group, associated with physical, psychological, spiritual, and social health education and promotion.

### 5.1 Limitations

The main limitation of this study was that the 2 groups used were limited to 54 third-year nursing students in a 5-year program as an experimental group and 56 third-year nursing students in a 5-year program as a control group. Specifically, there was no appropriate placebo control for the control group. All of the participants were limited to nursing students with data collection at the Department of Nursing in a university in Kaohsiung City, Taiwan R.O.C.

## 6 Conclusions

The current study demonstrated that nursing students in the experimental and control groups exhibited significant differences in meaning of life, positive beliefs, and well-being on the pre-post-test and pre-post post-test. Nursing students in the experimental group achieved significant improvement on the immediate and delayed effects in meaning of life, positive beliefs and well-being, thereby enhancing physical, psychological, spiritual, and social health education and promotion.

## Author contributions

**Conceptualization:** Fu-Ju Tsai, Yih-Jin Hu, Cheng-Yu Chen.

**Data curation:** Fu-Ju Tsai, Yih-Jin Hu.

**Formal analysis:** Fu-Ju Tsai, Yih-Jin Hu, Cheng-Yu Chen.

**Funding acquisition:** Fu-Ju Tsai.

**Investigation:** Fu-Ju Tsai.

**Methodology:** Fu-Ju Tsai, Yih-Jin Hu, Cheng-Yu Chen.

**Project administration:** Yih-Jin Hu.

**Resources:** Fu-Ju Tsai.

**Software:** Fu-Ju Tsai.

**Supervision:** Yih-Jin Hu, Cheng-Yu Chen, Gwo-Liang Yeh, Chie-Chien Tseng, Si-Chi Chen.

**Validation:** Yih-Jin Hu, Cheng-Yu Chen, Gwo-Liang Yeh, Chie-Chien Tseng, Si-Chi Chen.

**Visualization:** Yih-Jin Hu, Cheng-Yu Chen, Gwo-Liang Yeh, Chie-Chien Tseng, Si-Chi Chen.

**Writing – original draft:** Fu-Ju Tsai.
